# The role of mitochondria in longevity and healthspan

**DOI:** 10.1186/2046-2395-3-7

**Published:** 2014-05-22

**Authors:** Martin D Brand

**Affiliations:** 1 The Buck Institute for Research on Aging, 8001 Redwood Blvd, Novato, CA 94945, USA

## Abstract

The role of mitochondria in aging and disease remains contentious more than 40 years after the mitochondrial free radical theory of aging was first proposed. As part of a wider cross-journal series on contemporary mitochondrial biology, *Longevity & Healthspan* presents a thematic series of four reviews that discuss the evidence for and against the modern incarnations of the theory, and examine the relevance of mitochondrial membrane phospholipid unsaturation and the interactions of mitochondria with sex hormones.

## Editorial

Mitochondria are indispensable for aerobic life. Depending on the tissue and species, they normally occupy 2-20% of the volume of a cell, and it can be up to 50%. They have their own DNA and undergo constant motion, fusion and fission. They provide the cell with ATP formed during the oxidation of carbohydrate, proteins and fats and are involved in a range of metabolic pathways including gluconeogenesis, urea synthesis, one-carbon metabolism, protein synthesis, amino acid metabolism, haem synthesis and hormone synthesis. They help regulate cellular calcium levels, trigger apoptosis, and can generate reactive oxygen species that are used in cellular signalling and can cause oxidative damage. Their many different roles are highlighted in a cross-journal thematic series including not only articles in *Longevity & Healthspan*, but also in *Extreme Physiology & Medicine*, *Cancer & Metabolism* and *BMC Biology*.

The hallmark of a useful hypothesis is that it stimulates further work and drives progress in its field. The free radical theory of aging has certainly done that since Harman proposed it in its general form in 1956 [1] and in a more mitochondrially-oriented form in 1972 [2]. This theory proposes that the primary cause of aging is mitochondrial production of free radicals and the mitochondrial damage that ensues. The evidence that has been gathered over the years has led to adjustments and refinements in the formulation of the hypothesis as different authors have attempted to articulate it more precisely and to square it with experimental observations; as a result there have been numerous updates and overviews [3-6]. Many of these have been very influential – I remember first reading the seminal review by Beckman and Ames [7] and being impressed by the breadth of the circumstantial evidence that they highlighted. However, the perspective has become increasingly critical as the predicted beneficial effects of many antioxidant treatments and genetic manipulations have failed to materialise [8-14].

Where does that leave us today? On the one hand, many of the manipulations that should decrease aging and increase longevity according to the classical versions of the mitochondrial free radical theory of aging have failed to do so [15-18], implying that the theory is wrong, or at best deeply flawed. On the other hand, some of these manipulations have been very successful [19-22], implying that that the theory refracts some underlying reality and still has significant value as a guide to thought and experiment.

In this thematic series on the role of mitochondria in longevity and healthspan, Dai *et al*. [23] discuss the evidence relating to the free radical theory with an emphasis on results that are generally supportive. They review studies that support the role of mitochondrial oxidative stress and dysfunction in aging and healthspan, including cardiac aging, age-dependent cardiovascular diseases, skeletal muscle aging, neurodegenerative diseases, insulin resistance, diabetes and age-related cancers, then consider potential drugs to improve mitochondrial function in aging and healthspan.

Stuart *et al*. [24] take the opposite tack, examining the evidence from long- and short-lived animal species, calorie restriction and genetically modified animals. They argue that the mitochondrial free radical theory of aging is in crisis because recent studies have shown no relationship between free radicals and longevity, and instead emphasize a role for mitochondrial reactive oxygen species as intracellular messengers. They propose that if mitochondrial reactive oxygen species are involved in aging and longevity, it is through specific and regulated cellular processes and not through indiscriminate oxidative damage.

An important mechanism that may link reactive oxygen species to cellular phenotypes is the oxidation of polyunsaturated fatty acyl groups in membrane phospholipids [25-27]. This topic is discussed by Valencak and Azzu [28], who review the many published correlations between mitochondrial membrane phospholipid composition and longevity. Although there are methodological issues, the ratio of n-3:n-6 polyunsaturated fatty acyl groups does appear to correlate with lifespan. However, they caution against simply relating these two traits because correlation is consistent with but does not prove a functional relationship.

One aspect of mitochondria and healthspan that has received less attention until recently is the role of sex steroid hormones. This is addressed by Velarde [29], who discusses the evidence that poorer healthspan is associated with declining circulating levels of these hormones [30-32]. Mitochondria are involved in steroid hormone synthesis, and in turn mitochondrial function may respond to sex hormones. He suggests that a decline in sex steroid hormones and accumulation of mitochondrial damage creates a positive feedback loop that may contribute to progressive degeneration in aging.

Together this set of four reviews illuminates several aspects of our contemporary understanding of the role of mitochondria in healthspan and longevity and should help to light the path ahead. Further unsolicited articles are encouraged to the series and should be submitted via the journal’s online submission system.
